# Genomics and lipidomics analysis of the biotechnologically important oleaginous red yeast *Rhodotorula glutinis* ZHK provides new insights into its lipid and carotenoid metabolism

**DOI:** 10.1186/s12864-020-07244-z

**Published:** 2020-11-26

**Authors:** Chun-Ji Li, Die Zhao, Ping Cheng, Li Zheng, Guo-Hui Yu

**Affiliations:** 1 Innovative Institute for Plant Health, Zhongkai University of Agriculture and Engineering, Guangzhou, 510225 People’s Republic of China; 2 College of Agriculture and Biology, Zhongkai University of Agriculture and Engineering, Guangzhou, 510225 People’s Republic of China; 3 National Key Laboratory of Crop Genetic Improvement, Huazhong Agricultural University, Wuhan, 430070 People’s Republic of China

**Keywords:** *Rhodotorula glutinis*, Lipids, Carotenoids, Genome assembly, Lipidomics

## Abstract

**Background:**

*Rhodotorula glutinis* is recognized as a biotechnologically important oleaginous red yeast, which synthesizes numerous meritorious compounds with wide industrial usages. One of the most notable properties of *R. glutinis* is the formation of intracellular lipid droplets full of carotenoids. However, the basic genomic features that underlie the biosynthesis of these valuable compounds in *R. glutinis* have not been fully documented. To reveal the biotechnological potential of *R. glutinis*, the genomics and lipidomics analysis was performed through the Next-Generation Sequencing and HPLC-MS-based metabolomics technologies.

**Results:**

Here, we firstly assemble the genome of *R. glutinis* ZHK into 21.8 Mb, containing 30 scaffolds and 6774 predicted genes with a N50 length of 14, 66,672 bp and GC content of 67.8%. Genome completeness assessment (BUSCO alignment: 95.3%) indicated the genome assembly with a high-quality features. According to the functional annotation of the genome, we predicted several key genes involved in lipids and carotenoids metabolism as well as certain industrial enzymes biosynthesis. Comparative genomics results suggested that most of orthologous genes have underwent the strong purifying selection within the five *Rhodotorula* species, especially genes responsible for carotenoids biosynthesis. Furthermore, a total of 982 lipids were identified using the lipidomics approaches, mainly including triacylglycerols, diacylglyceryltrimethylhomo-ser and phosphatidylethanolamine.

**Conclusion:**

Using whole genome shotgun sequencing, we comprehensively analyzed the genome of *R. glutinis* and predicted several key genes involved in lipids and carotenoids metabolism. By performing comparative genomic analysis, we show that most of the ortholog genes have undergone strong purifying selection within the five *Rhodotorula* species. Furthermore, we identified 982 lipid species using lipidomic approaches. These results provided valuable resources to further advance biotechnological applications of *R .glutinis*.

**Supplementary Information:**

The online version contains supplementary material available at 10.1186/s12864-020-07244-z.

## Background

*Rhodotorula* is a genus of oleaginous pigmented yeasts, part of the phylum Basidiomycota, class Microbotryomycetes, and order Sporidiobolales [1]. *R. glutinis* is recognized as a representative species of genus *Rhodotorula*, and it was reported firstly by F.C. Harrison in 1928 [2]. This species grows on a broad spectrum of ecological environments, ranging from air, soil, and ocean, as well as in the bodies of animals, plants, and lower organisms [3]. Most of these strains are aerobic, mesophilic, and spherical, ellipsoidal, or elongated in shape [4]. *R. glutinis* is of great industrial importance as it synthesizes numerous valuable compounds, such as, lipids (SCO, single-cell oils), carotenoids, and enzymes [5, 6].

*R. glutinis* strains are exceptionally robust in converting low-cost carbohydrates into lipids, such as triglycerides (TAGs) [7]. This species also produces several microbial oils, mainly including oleic, linoleic, palmitic, and stearic acid [8, 9]. These lipid metabolites account for up to 72% of its cells dry mass, and could be used as food additives, diet supplements and raw material to produce second-generation biodiesel [10]. Depending on growth conditions, its colony color presents yellow, pink, and blood red, largely due to the relative proportion of the carotenoids produced, including torulene, torularhodin, and β-carotene [11, 12]. Due to their health-promoting properties, these carotenoids are of great biotechnological potential could be used in the food, pharmaceutical, cosmetics and feed industries [13]. Previous studies concluded that both of torulene and torularhodin possess stronger anti-oxidative properties than β-carotene [14, 15]. Furthermore, the growing scientific evidences suggested that torulene and torularhodin may have potential benefits in the prevention of tumors, especially prostate and liver cancer [16–18]. Additionally, torularhodin is capable of enhancing the antimicrobial ability of TiO2/Ti materials, and it may become a novel natural antimicrobial agent [19, 20]. Torularhodin supplementations significantly alleviates the alcoholic liver disease via decreasing the levels of ethanol-induced aspartate transaminase (ALT), aspartate transaminase (AST) and low density lipoprotein (LDL) [21]. Due to its higher biosynthetic efficiency, *R. glutinis* has been considered as one of the most important producers of torulene and torularhodin, especially the latter [22, 23]*.* The toxicological evaluation concluded that the pigments torulene and torularhodin extract from *R. glutinis* yeasts could be used safely as food additives, which retain anti-oxidative properties as well as colorant role [24].

Besides, biomass of *R. glutinis* strains could be used as the natural sources of lipases, α-L-arabinofuranosidase, invertase, pectinases, and tannin acyl hydrolase, particularly phenylalanine ammonia lyase (PAL) [25, 26]. Furthermore, *R. glutinis* has been regarded as a biocontrol agent for post-harvest microbial diseases of fruit [27], possibly due to its antagonistic attachment capability to pathogenic bacteria and production of torularhodin [28, 29]. More importantly, lipids, carotenoids and industrial enzymes synthesized by *R. glutinis* strains have advantages over others, mainly due to a higher biotransformation rate, low-costs and independent of climates [30, 31]. Therefore, the usage of *R. glutinis* strains as bioreactors for the production of industrial bio-products using low-cost natural substrates have given rise to a strong interest currently [32].

Nevertheless, despite a considerable number of literatures presenting the industrial applications of *R. glutinis* strains, little is known about its basic genomic characteristics (formerly *R. glutinis* ATCC 204091 has been designated as *R. toruloides*) [33, 34]. Genetic backgrounds involved in the biosynthesis of lipids, carotenoids, enzymes and other precious metabolites in *R. glutinis* still remain poorly studied. The PacBio single-molecule long-read sequencing [35] and LC–MS/MS-based lipidomics approaches offer the advantage of increased precision of genome assembly and lipid metabolites identification [36]. Here, to better enable the continued industrial applications of this versatile single-cell yeast, we present the *de novo* genome assembly and shotgun lipidomics evaluation of the *R. glutinis* strain ZHK. We also qualitatively and quantitatively determined the carotenoid contents in the strain ZHK. Subsequently, we carried out a comparative analysis to investigate orthologous and species-specific genes between *R. glutinis* and its closely related species. Through comparative genome analysis of *R. glutinis*, we explored the evolutionary dynamics with its close related *Rhodotorula* species. Continuously, this work significantly enriched our understanding of the molecular basis underlying the industrial bio-products synthesis of this species. This should promote the development of the genetic engineering for the overproducing of bioactive natural products in *R. glutinis* strains.

## Results

### Genome sequencing, assembly and assessment

**Fig. 1:**
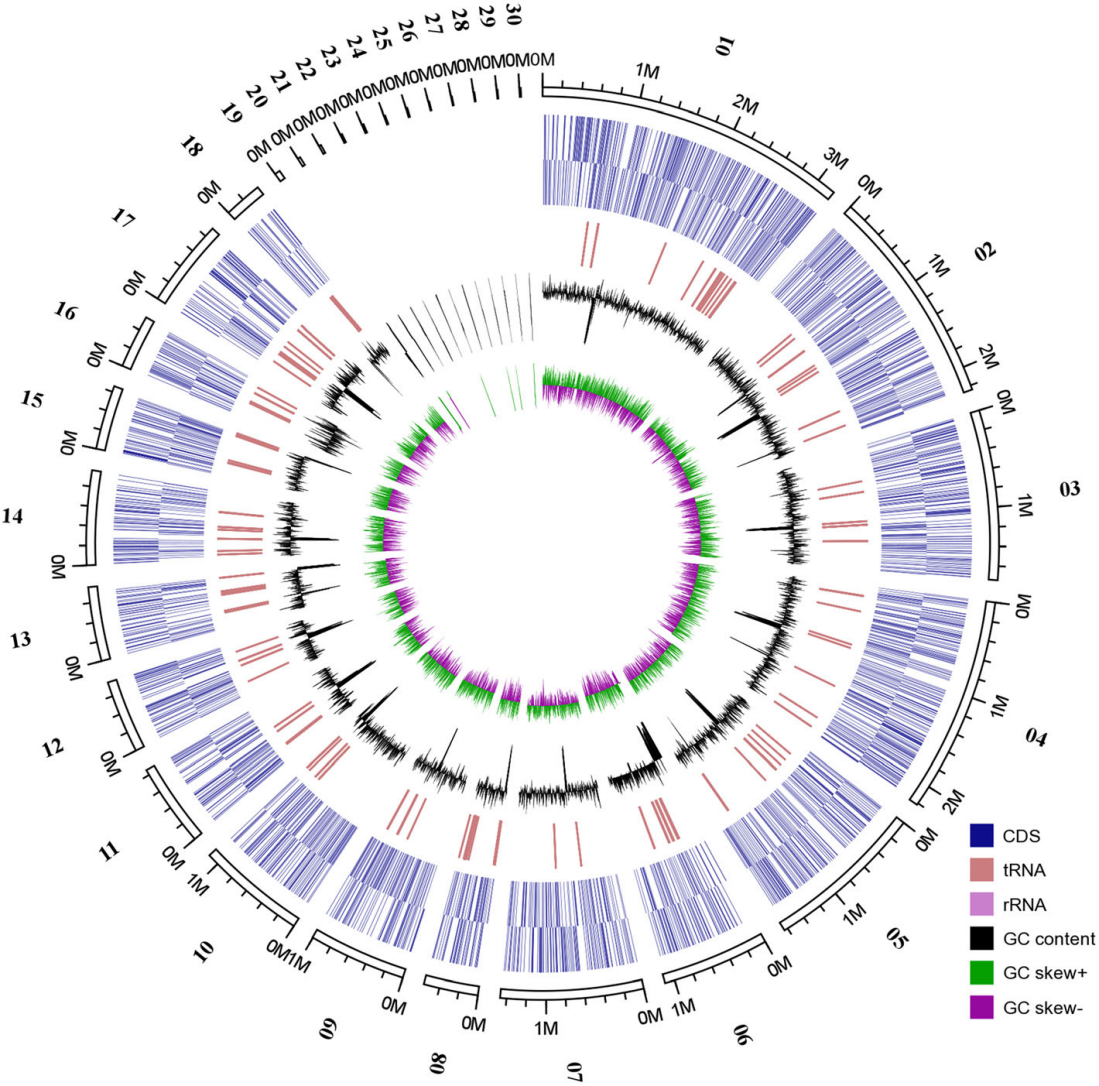
Genomic features of *R. glutinis* strain ZHK. The 21.8 Mb genome of strain ZHK, containing 30 scaffolds, the longest length scaffold of 3,195,425 bp, the shortest length scaffold of 10,281 bp, a N50 length of 1,466,672 bp, a GC content of 67.8%. The ZHK genome encodes 6774 predicted proteins and 156 transfer RNAs (tRNAs), which are validated by RNA-seq. From the outer circle to the inner, it represents the length of scaffolds, coding sequence (CDS), tRNAs, GC content (black) and GC skew curve (Green: positive GC skew; Violet: negative GC skew), respectively

Here, the whole genome shotgun sequencing of oleaginous red yeast *R. glutinis* ZHK was accomplished using the PacBio Sequel system with a Single Molecule Real-Time (SMRT) sequencer and an Illumina Hiseq 2500 system. As a result, a total of 6.62 Gb polymerase reads from a 20 kb library was generated by SMRT sequencing. After removing adapters and low-quality reads, we obtained 6.57 Gb (~300×) subreads for whole genome assembly. Primary contigs were assembled and corrected from PacBio long reads using the program MECAT. The obtained contigs were further polished using 3.6 Gb (~170×) short paired-end reads (PE150, Illunima) by the program Pilon. As shown in Fig. 1, our final assembled genome of the strain *R. glutinis* ZHK consists of 30 scaffolds, a N50 length of 1,466,672 bp, the longest length scaffold of 3,195,425 bp, the shortest length scaffold of 10,281 bp, a GC content of 67.8%, and a size of 21.8 Mb. Using the program GeneMark-ES, we predicted 6774 genes in the ZHK genome with an average length of 1813 bp and a mean GC content of 69.59%, which occupies 55.0% of the whole genome sequence. In addition, a total of 156 transfer RNAs (tRNAs) were identified in the ZHK genome using the program tRNAscan-SE, with an average length of 90 bp. The full-length tRNAs totally comprised 14053 bp, accounting for 0.06% of the whole genome sequence. The results of the BUSCO alignment showed that the assembled genome contains 1272 complete BUSCOs (94.4%), of which 1260 were single-copy, while 12 were duplicated (Additional file 1). For the transcriptome sequencing (RNA-seq) results, a total of 3.54 Gb raw reads (Q20: 95%, Q30: 90%, and GC-content: 66.32%) were generated. Regarding the results of RNA-seq, we found that 6642 (98.05%) genes predicted in the ZHK genome regions and 143 novel genes were expressed (Additional file 2). Furthermore, the RNA-seq results showed that 93.92% reads were matched to exon regions, 2.18% to intron regions, and 3.91% to intergenic regions. These reads are aligned to the intron region, possibly due to the intron retention or alternative splicing events. In addition, a total of 1513 SNPs/InDel (Additional file 3) were identified through comparing RNA-seq data with the ZHK whole genome sequence. From the RNA-seq results, we also obtained the boundaries of 5’UTR and 3’UTR of 1814 predicted genes in the ZHK genome (Additional file 4). Consequently, both of BUSCO alignment and transcriptome mapping indicated that our current genome assembly with a high-quality of completeness. Up to now, except for our *R. glutinis* ZHK, there are eleven whole genome sequences of *Rhodotorula* species have been sequenced. In general, the genome size of these *Rhodotorula* species is ~20 Mb, which represents a smaller grade when compared to other classes in order Pucciniomycotina [37]. The phenomenon of the decrease in genome size is considered as a shared feature in class Microbotryomycetes.

### Functional annotation

As for the 6774 predicted genes, 6560 (96.84%) genes could be annotated using NCBI Nr databases based on sequence homology. Among these genes annotated to Nr database, the top three species of matched gene number are *Rhodotorula toruloides* NP11 (3141, 47.88%), *Rhodotorula graminis* WP1 (521, 7.94%), and *Rhodotorula* sp. JG-1b (479, 7.30%). Moreover, 3831 (56.55%), 3345 (50.12%), and 2880 (42.52%) genes could be annotated according to the Swissport, KOG and KEGG databases, respectively. Of which, these genes assigned to KOG categories, are involved in 31082 proteins and domains. Top five KOG categories of annotated gene number are “General function prediction only (799, 23.88%)”, “Posttranslational modification, protein turnover, chaperones (689, 20.59%)”, “Signal transduction mechanisms (640, 19.13%)”, “RNA processing and modification (406, 12.13%)”, and “Energy production and conversion (380, 11.36%)”. In order to further understand the coding genes of candidate for biotechnological potential in the ZHK genome, the 2880 genes have been successfully assigned to their orthologous in the KEGG Pathways database. Of which, there are 1609 genes have been enriched in 21 categories of KEGG_B_class, including 120 KEGG pathways. Top three KEGG pathways of annotated genes number are “Biosynthesis of antibiotics (ko01130; 200, 12.43%)” (Additional file 5), “Biosynthesis of amino acids (ko01230; 100, 6.22%)”, and “Carbon metabolism (ko01200; 85, 5.28%)”. Based on the KEGG pathways mapping, we annotated several candidate genes for biotechnological potential as following: 1) lipid metabolism, including genes encoding ACACa (acetyl-CoA carboxylase), ACOX3 (acyl-CoA oxidase), and PDAT (phospholipid:diacylglycerol acyltransferase); 2) biosynthesis of carotenoids, including *crtE* (geranylgeranyl diphosphate synthase), *crtI* (phytoene desaturase), and *crtYB* (bifunctional, phytoene synthase/lycopene beta-cyclase); 3) biosynthesis of enzymes, including genes encoding PAL (phenylalanine ammonia-lyase), TGL2 (triacylglycerol lipase), and MGLL (acylglycerol lipase).

**Fig. 2:**
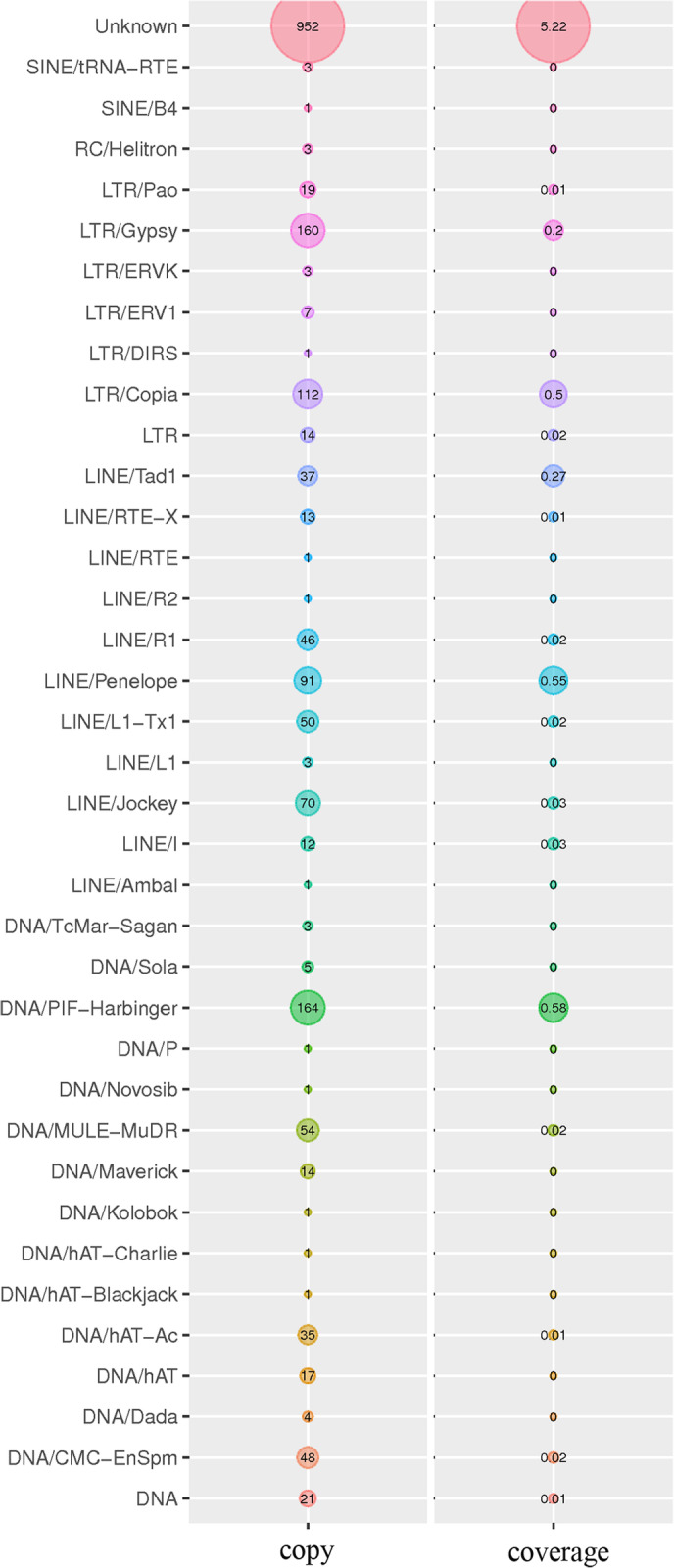
Distribution of transposable element (TE) families in the assembled *R. glutinis* strain ZHK genome. Copy: total copy number per TE family; Coverage: TE coverage (%) in whole genome assemblies. The TE types and TE family names are listed at left

Furthermore, there are 1069 predicted genes could be classified into three Gene Ontology (GO) categories: Cellular Component (581 genes), Biological Process (812 genes), and Molecular Function (657 genes). These 1069 genes mainly distributed across five functional entries, including “Cellular Process (558, 52.20%)”, “Metabolic Process (555, 51.92%)”, “Cell (512, 47.90%)”, “Catalytic Activity (467, 43.69%)”, and “Single-organism Process (421, 39.38%)”. In addition, we identified 43 interspersed repetitive sequences and 6348 tandem repeats, including 103 macro-satellites DNA, 2870 mini-satellite DNA and 1234 micro-satellite DNA in the ZHK genome. Moreover, a total of 1,599,119 bp full-length TEs were predicted in the ZHK genome. As shown in Fig. 2, these TEs mainly include 316 (0.73%) LTR-REs, 325 (0.93%) LINE-Res, 370 (0.64%) DNA transposons, 325 LINE-REs and 952 (5.22%) unknowns, of which 16.04% are Class LTR element, mainly assigned to Copia (112), Gypsy (160) and Pao (19). The full-length TEs totally accounts for 7.16% of the ZHK whole genome sequence.

### Analysis of lipidomics and carotenoids

In order to investigate the lipid metabolites in *R. glutinis* ZHK cell, we performed the shotgun lipidomics profiling for qualitative and quantitative characterization of lipidome in mixed samples acquired from three growth periods (24 h, 48h, and 72h). Samples were processed for chromatographic separation and mass spectrometry. As shown in Table 1, a total of 982 lipid species were identified using data-dependent MS/MS scans in both positive (POS) and negative (NEG) mode, mainly including 325 Triacylglycerols (TAGs), 95 Diacylglyceryltrimethylhomo-Ser (DGTS), 64 Phosphatidylethanolamine (PE), 62 Phosphatidylcholine (PC), 64 Ceramide (Cer), 40 Hexosylceramide (HexCer), 35 Sulfoquinovosyl-diacylglycerol (SQDG), 31 Glucuronosyldiacylglycerol (GlcADG), 27 Diacylglycerols (DAG), 26 Sulfatide (SHexCer), 26 Phosphatidylinositol (PI) and 23 Fatty acid (FA) (Comprehensive information of all identified lipid metabolites is given in Additional file 6). To gather further information of carotenoids biosynthesis in *R. glutinis* ZHK, we employed the HPLC analysis to evaluate carotenoid contents in the same mixed samples with lipidomics profiling, both qualitatively and quantitatively. It is shown that the total amount of carotenoids in *R. glutinis* ZHK reaches to 1276.02 μg/g_dw_. The relative proportion of torularhodin, torulene and β-carotene of total carotenoids accounts for 66.64% (890.25 μg/g_dw_), 14.07% (179.51 μg/g_dw_), and 19.29% (206.36 μg/g_dw_), respectively.

**Table 1:** Lipid metabolites were identified using shotgun lipidomics profiling in *R. glutinis* ZHK

Lipids name	POS	NEG	Percentage (%)
Triacylglycerols (TAGs)	325	–	33.09
Diacylglyceryltrimethylhomo-Ser (DGTS)	95	–	9.67
Phosphatidylcholine (PC)	38	24	6.31
Phosphatidylethanolamine (PE)	28	36	6.52
Diacylglycerols (DAG)	27	–	2.75
Apocarotenoids (ACar)	20	–	2.04
Phosphatidylmethanol (PMeOH)	8	3	1.12
Phosphatidyl glycerol (PG)	6	12	1.83
Sulfatide (SHexCer)	3	23	2.65
Phosphatidylserine (PS)	3	6	0.92
Lysophosphatide (LPE)	3	3	0.61
Lyso-phosphatidylcholine (LPC)	2	2	0.4
Hexosylceramide (HexCer)	2	40	4.28
Ceramide (Cer)	4	60	6.52
Sulphoquinovosyldiglyceride (SQDG)	–	35	3.56
Glucuronosyldiacylglycerol (GlcADG)	–	31	3.16
Phosphatidylinositol (PI)	–	26	2.65
Fatty acids (FA)	–	23	2.34
Phosphatidic acid (PA)	–	16	1.63
fatty acid esters of hydroxyl fatty acids (FAHFA)	–	14	1.43
Oxidized phosphatidylethanolamine (OxPE)	–	8	0.81
Acyl-Glucuronosyldiacylglycerol (AcylGlcADG)	–	6	0.61
Oxidized phosphatidylinositol (OxPI)	–	5	0.5
Sphingomyelin (SM)	–	3	0.31
Ganglioside (GM3)	–	3	0.31
Oxidized phosphatidyl glycerol (OxPG)	–	2	0.2
Others	30	7	3.78
Total	594	388	100

### Analysis of phylogenetic and syntenic relationships

**Fig. 3:**
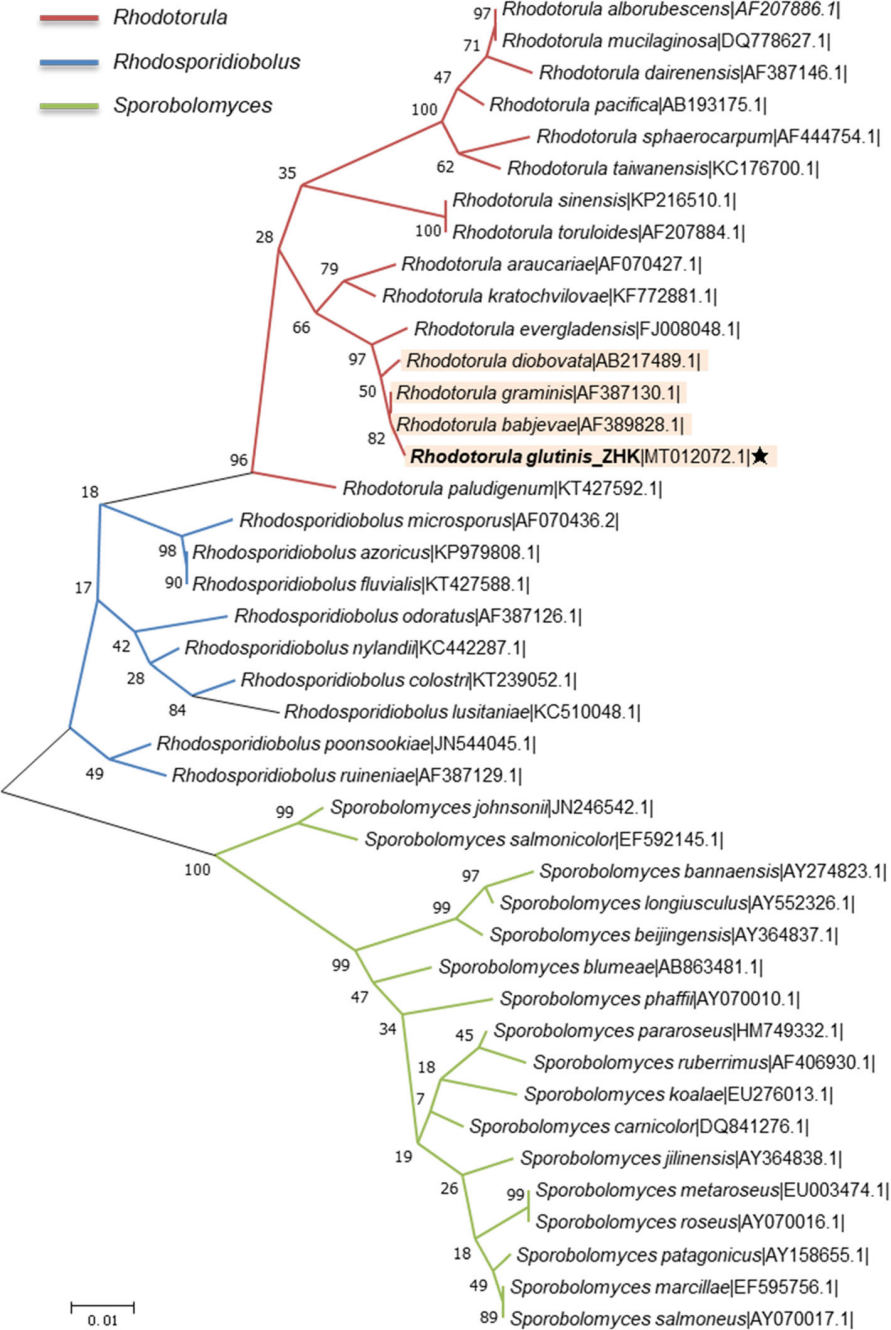
The phylogeny and evolutionary analysis between the 42 red yeast species of order Sporidiobolales. The phylogenetic tree was constructed using the software MEGA 7.0 based on the alignment of the 26S rDNA sequences combined with the Neighbor-joining method and Bootstrap analysis of 1000 replicates. The strain *R. glutinis* ZHK front is marked in bold. The strain ZHK and its closely related species are highlighted with pink area. The number at each branch of phylogenetic tree indicates the bootstrap value (1000 replicates). Red, blue, and green lines indicate the different clusters of *Rhodotorula*, *Rhodosporidiobolus* and *Sporobolomyces* isolates, respectively

**Fig. 4:**
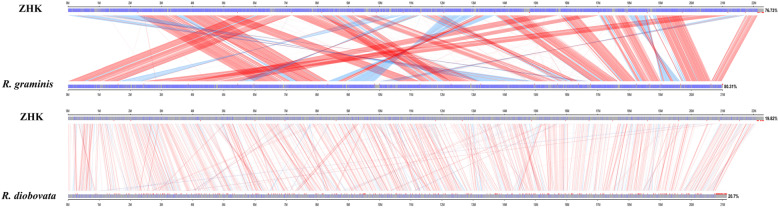
Syntenic relationships between *R. glutinis* ZHK genes with its two close related species. Pair-wise alignments between whole genome sequences of *R. glutinis* ZHK, *R. graminis* and *R. diobovata* were performed using the program MCScanX. Red lines represent collinear blocks of similarity, while the blue bars indicate the collinear blocks of reverse compliment in two genomes

Taxonomically, a total of 42 yeast species have been accepted in order Sporidiobolales (*Rhodosporidiobolus*: 9; *Rhodotorula*: 15; *Sporobolomyces*: 18) [38]. These strains are commonly known as the oleaginous red yeasts because of their synthesis of lipid droplets full of carotenoids. In order to investigate the phylogenetic relationships between theses oleaginous red yeasts assigned to order Sporidiobolales, we constructed the phylogenetic tree with the available 26S rDNA sequences form NCBI Nucleotide database. As shown in Fig. 3, as for the genus *Rhodotorula*, the strain *R. glutinis* ZHK showed a closer evolutionary relationship with *R. babjevae*, *R. graminis* and *R. diobovata* than the other species. Basing on the results of phylogenetic analysis, we carried out the syntenic analysis of genome between *R. glutinis* ZHK with its closely related species with available genome sequence, namely *R. graminis* and *R. diobovata*. As shown in Fig. 4, the results revealed that the ZHK genome situates a higher syntenic relationship with *R. graminis* than *R. diobovata*. There are 9737 collinear blocks between the whole genome sequence of *R. glutinis* ZHK and *R. graminis,* the proportion of the total base length in collinear blocks account for their total gene length are higher than 75%. Moreover, a total of 11413 collinear blocks was identified between *R. glutinis* ZHK and *R. diobovata*. The full length of collinear blocks accounts for 19.82% and 20.7% in the whole genome of *R. glutinis* ZHK and *R. diobovata,* respectively.

### Comparative analysis of protein families and genes

**Fig. 5:**
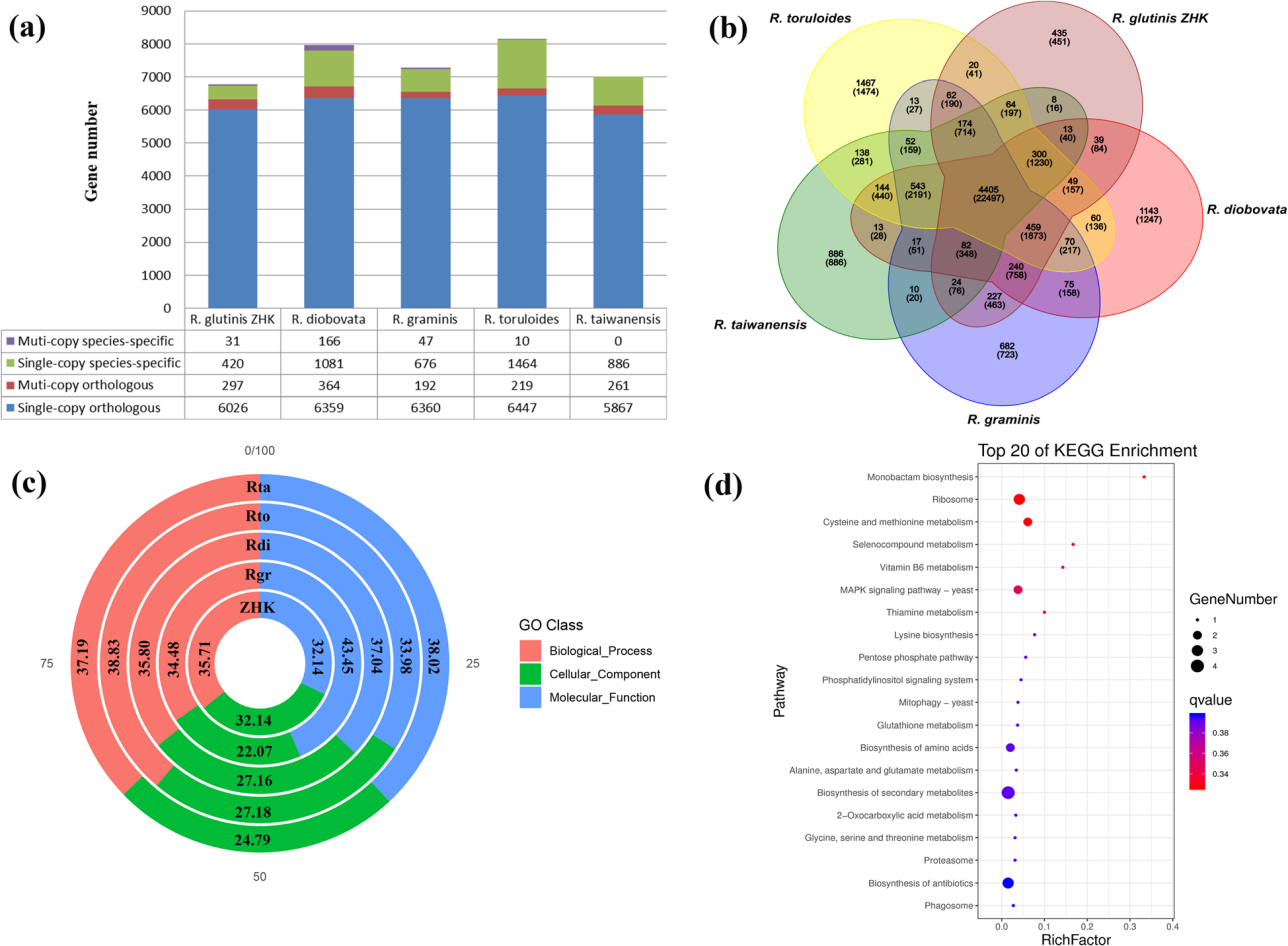
Comparative genomic analysis of *R. glutinis* ZHK with four related species with available genome sequence, namely *R. graminis*, *R. diobovata, R. toruloides and R. taiwanensis*. **(a)** Distribution of single /multi-copy orthologous and species-specific genes among five *Rhodotorula* species. **(b)** Venn diagram showing the shared/unique genes in *R. glutinis* ZHK and comparison with those in *R. graminis*, *R. diobovata, R. toruloides and R. taiwanensis*, respectively. **(c)** Relative proportion (%) of the genes of species-specific protein families enriched to different GO categories, in five genomes of five *Rhodotorula* species, respectively. **(d)** Top 20 enriched KEGG pathways of these species-specific genes in *R. glutinis* ZHK whole genome. Rich factor indicates the ratio of the enriched genes number to the total gene number in a certain pathway. The Q-value results from the *p*-value via multi-test correction. The ranges of Q-value are from 0 to 1 and a higher enrichment is achieved when the Q-value reaches to 0

Here, a total of 6774 protein-coding genes have been predicted in the ZHK genome, and the top four *Rhodotorula* species of these protein-coding genes annotated into Nr database were *R. toruloides*, *R. graminis*, *R. diobovata* and *R. taiwanensis.* Hence, we performed a comparative genomics analysis between *R. glutinis* ZHK with the four relative closely related species. As shown in Fig. 5a, we compared the distribution of predicted genes among the five oleaginous red yeasts. To investigate the species-specific gene/protein families, the pairwise comparisons have been carried out via a series of BLASTX searches within the five oleaginous red yeasts. As shown in Fig. 5b, we identified 11915 protein families regarding the similarities between gene sequences from the five yeast species (6601 families for *R. glutinis* ZHK, 8020 families for *R. toruloides*, 7135 families for *R. graminis*, 7652 families for *R. diobovata,* and 6873 families for *R. taiwanensis*). Of which, 435 (451 genes), 1467 (1474 genes), 682 (723 genes), 1143 (1247 genes), and 886 (886 genes) protein families were species-specific in *R. glutinis* ZHK, *R. toruloides*, *R. graminis*, *R. diobovata* and *R. taiwanensis*, respectively.

In order to better understand the functional classification of these species-specific genes, we conducted the GO and KEGG enrichment analysis. As shown in Fig. 5c, we performed the GO analysis using their respective species-specific genes of the five oleaginous red yeasts. Among the species-specific genes of the *R. glutinis* ZHK (Additional file 7), 49 (37.98%), 26 (20.16%) and 54 (41.86%) GO terms were enriched in three categories Biological Process (BP), Molecular Function (MF), and Cellular Component (CC), respectively. It was found that the significantly enriched GO terms of the species-specific genes in *R. glutinis* ZHK, including BP: metabolic process (GO:0008152), organic substance metabolic process (GO:0071704), cellular process (GO:0009987), single-organism cellular process (GO:0044763) and single-organism process (GO:0044699); MF: catalytic activity (GO:0003824), binding (GO:0005488), hydrolase activity (GO:0016787), small molecule binding (GO:0036094), and heterocyclic compound binding (GO:1901363); CC: intracellular (GO:0005622), cell (GO:0005623), organelle (GO:0043226), membrane (GO:0016020), and membrane-bounded organelle (GO:0043227). After that, we carried out the KEGG pathway mapping using the species-specific genes of the *R. glutinis* ZHK. As shown in Fig. 5d, the significantly enriched KEGG pathways of the species-specific genes in *R. glutinis* ZHK mainly contain biosynthesis of secondary metabolites (ko01110), ribosome (ko03010), biosynthesis of antibiotics (ko01130), cysteine and methionine metabolism (ko00270), MAPK signaling pathway–yeast (ko04011), and biosynthesis of amino acids (ko01230).

In order to investigate the evolutionary dynamics, we identified 3952 one-to-one orthologous genes between *R. glutinis* ZHK and its four closely related *Rhodotorula* species (Additional file 8). The *Ka* and *Ks* calculation of the single-copy orthologous genes is widely regarded as an indicator of selective pressure during biological evolution [39]. In order to evaluate the general variation in the selective restriction within the five *Rhodotorula* species at gene levels, we calculated the substitution rate (*Ka*/*Ks*) for each orthologous gene using the free ratio model. The results showed that the most of these orthologous genes exhibit a relative low substitution rate (*Ka*/*Ks* < 0.5), which indicates that these orthologous genes have been retained via a series of strong purifying natural selection. Of which, we found that there are 1 pairs with a *Ka*/*Ks* value > 1.0 (strong positive selection), 3 pairs with a *Ka/Ks* value between 0.5 and 1.0 (positive selection), 621 pairs with a *Ka/Ks* value between 0.1 and 0.5 (weak positive selection), and 3327 pairs with a *Ka/Ks* value < 0.1 (purifying selection). The categories of KEGG_B_class enriched among the positively selected genes contain “Amino acid metabolism”, “Folding, sorting and degradation”, and “Translation”. Meanwhile, the categories of KEGG_B_class enriched among the purifying selected genes mainly include “Amino acid metabolism”, “Biosynthesis of other secondary metabolites”, “Carbohydrate metabolism”, “Energy metabolism”, “Folding, sorting and degradation”, “Glycan biosynthesis and metabolism”, “Lipid metabolism”, “Metabolism of cofactors and vitamins”, “Metabolism of terpenoids and polyketides”, “Transport and catabolism”, “Translation”, “Signal transduction”, “Replication and repair”, and “Nucleotide metabolism” (Additional file 9).

## Discussion

*R. glutinis*, one of the most representative species of genus *Rhodotorula*, was recognized as a ubiquitous yeast ranging from contrasting ecosystems such as marine, soil, lake, plant leaf and even polar ice. *R. glutinis* is versatile oleaginous red yeast capable of producing several valuable compounds including microbial lipids, carotenoids and enzymes and therefore has been regarded as a promising host for bio-refinery. Notwithstanding a considerable amount of literature has documented the industrial applications of lipids, carotenoids and enzymes production by *R. glutinis*, and the molecular basis unraveling the biosynthetic mechanism of these valuable compounds still remains largely limited. Mainly because the genomics backgrounds of this species has not yet been studied. In addition, the understanding of the lipid composition is of significant foundation to genetically enhance microbial oils. The advanced Next-Generation Sequencing and HPLC-MS-based metabolomics technology have been widely used to study genetic and metabolic systems of microorganisms without available genome information.

Here, we firstly present the results of whole-genome sequencing and shotgun lipidomics profiling of *R. glutinis* ZHK, to identify its genomics features and intracellular lipid species. Lipids, generally including phospholipids, sphingolipids, fatty acids, sterols, and triacylglycerols (TAGs), are important biomolecules for all biological vitalities [40]. Currently, the issue of excessive consumption of non-renewable fuels has brought about some concerns of energy crisis and environmental pollution worldwide. Microbial lipids are also used as substrates in the third generation biodiesel feedstock, and possess advantages over the first and second generation biofuels [41]. Many oleaginous red yeasts of order Sporidiobolales produce lipids, especially TAGs [42]. TAGs, a group of neutral lipids, are usually used as the food additives, feed supplement and feedstock for chemical syntheses [43]. Oleaginous red yeasts are classified as strains that accumulate high lipids content, and therefore, considered as the potential oil resources for renewable biodiesel feedstock [42]. Furthermore, because of the advantages over the conventional resources, the lipids biosynthesis from oleaginous red yeasts has attracted increasing attention recently. Therefore, using metabolic engineering strategies to enhance lipids production is of great significance for economic and ecological sustainable development. These strategies are usually divided into following orientations that are directly or indirectly related to the biosynthesis of fatty acid and TAG [42]: 1) overexpression of key enzyme genes; 2) transcriptional regulation of bypass pathways; 3) restriction of competing pathways. As a type species of oleaginous yeast, *R. glutinis* is a robust platform organism for these lipids production, because it’s high biomass and multiple substrate availability. We found some candidate key enzyme genes which are involved in lipid metabolism in the ZHK genome. Some of key enzymes involved in the lipid metabolism, have been documented, such as ACC (acetyl-CoA carboxylase), AOX (acyl-CoA oxidase), PDAT (phospholipid: diacylglycerol acyltransferase), and GPDH (glycerol 3-phosphate dehydrogenase). ACC catalyzes acetyl-CoA to form malonyl-CoA; AOX catalyzing the first step in the pathway of fatty acid β-oxidation; PDAT catalyzes the acyl-CoA-independent synthesis of cholesterol esters; GPDH provides the activated glycerol backbone for TAG synthesis. Furthermore, we also found some genes related to lipases biosynthesis. Because of these lipases are closely related to lipid metabolism [44, 45], and therefore, also vital for the production of engineered lipid in *R. glutinis* ZHK. These genes could be the promising targets for genetic manipulation to enhance the production of lipid metabolites.

Additionally, the total carotenoid contents of *R. glutinis* ZHK were determined qualitatively and quantitatively. Torularhodin and torulene as the dominated carotenoid contents of *R. glutinis* ZHK collectively constitute 80.71% of total carotenoids, while the β-carotene only accounts for 19.29%. Torulene and torularhodin represent two of the principal carotenoids in *R. glutinis* strains and exhibit the similar chemical structure to that of super antioxidant lycopene. The earliest literature reported that the presence of torulene and torularhodin in microbial cells could be traced back to the 1930s and 1940s, respectively. But only in the last few decades, the amount of literature picturing their properties has gradually increased. Previous studies revealed that both of torularhodin and torulene possess considerable strong properties, such as anti-oxidative, anti-cancerous, anti-microbial and food safety. In order to realize the commercial application of these two carotenoids, it is essential to obtain highly efficient yeast strains of *R. glutinis*. However, until now, there have been no *R. glutinis* strains capable of producing torularhodin in high-yields required for industrial scale use. The rapid development of the gene editing and synthetic biology approaches allows us to construct an engineering strain that over-produces torularhodin and torulene. Moreover, the general pathways for carotenoid synthesis of oleaginous yeasts have proposed previously. However, the precise nature of the coding-genes involved in the bioconversion from torulene to torularhodin in these funguses still remains unclear. It is a bottleneck that currently blocks the industrial development of microbial torulene and torularhodin.

**Fig. 6:**
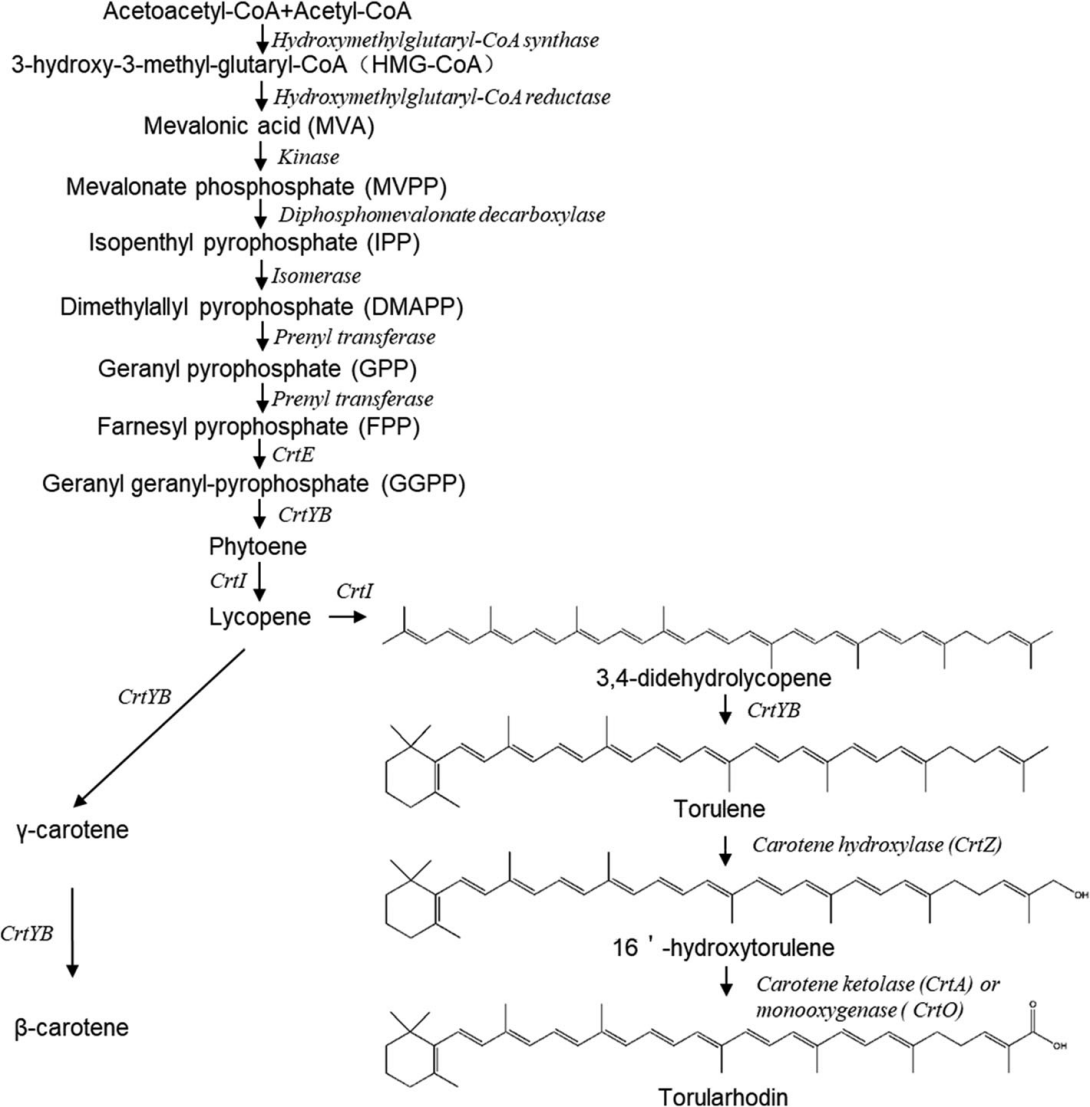
Schematic of the proposed biosynthetic pathways from acetyl-CoA to torularhodin and β-carotene in *R. glutinis* ZHK. These genes-encoding proteins annotated by orthology for enzymes which can catalyze pathway steps. Carotenogenic pathway: CrtE: GGPP synthase, CrtYB: bifunctional lycopene cyclase/phytoene synthase, CrtI: phytoene desaturase. Metabolites are shown in bold, while the enzymes responsible for relative bioconversions are indicated in italics at corresponding arrows

Generally, the bifunctional lycopene cyclase/phytoene synthase is capable of catalyzing 3,4-didehydrolycopene to form torulene in *Neurospora crassa* [46]. The 16’-hydroxytorulene could be regarded as an intermediate product of the biosynthetic pathway from torulene to torularhodin in the red yeasts *Cystofilobasidium infirmominiatum* [47]*.* Nevertheless, the enzymes involved in the transformation from torulene to torularhodin in this species have not been elucidated. Based on previous studies, as shown in Fig. 6, we propose the bioconversion process from torulene to torularhodin is as follows: 1) the carotene hydroxylase (CrtZ) catalyzes the hydroxylation of torulene to form 16’-hydroxytorulene; 2) the carotene ketolase (CrtA) or monooxygenase (CrtO) or both of them catalyzes the carboxylation of 16’-hydroxytorulene to form torularhodin. In the ZHK genome, we found some candidate genes (Table 2) putatively encoding GGPP synthase, lycopene cyclase/phytoene synthase, phytoene desaturase, hydroxylase, monooxygenase and ketolase, which may be related to the carotenoids biosynthesis. Interestingly, the comparative genomics results showed that these genes encoding GGPP synthase, phytoene desaturase and lycopene cyclase/phytoene synthase, has underwent the strong purifying natural selection within the five *Rhodotorula* species. Subsequently, we would like to further verify the function of these genes through a group of heterologous complementary experiments as described in our previous studies [48, 49]; hope to finally fill in the gaps in the synthetic pathway of torulene and torularhodin. Besides, the *R. glutinis* strains are regarded as the resources of various kinds of industrial enzymes, especially the phenylalanine ammonia lyase (PAL) [25]. The PAL is usually considered as the key limiting-enzyme in the biosynthesis of phenylpropanoids and flavones [50, 51]. Particularly, PAL has been used as an enzyme substitution therapy in medicine to treat phenylketonuria (PKU) [52]. Our genomics results reported here should be helpful for the further understanding the biosynthesis of PAL in *R. glutinis* ZHK. In brief, the present study will lay a theoretical foundation for increasing lipids, carotenoids, enzymes, and other bio-products production in *R. glutinis* strains, which would have an immense industrial significance and application.

**Table 2:** The candidate genes putatively encoding CrtE, CrtYB, CrtI, CrtZ, CrtO and CrtA in *R. glutinis* ZHK genome

Gene name	Gene ID	Description	Gene number
GGPP synthase	R_glutinis_G00415	*crtE*	1
lycopene cyclase/phytoene synthase	R_glutinis_G00924	*crtYB*	1
phytoene dehydrogenase	R_glutinis_G00921	*crtI*	1
hydroxylase	R_glutinis_G02426 R_glutinis_G04459 R_glutinis_G05828 R_glutinis_G04456 R_glutinis_G04508 R_glutinis_G01719	*crtZ*	6
monooxygenase	R_glutinis_G03787 R_glutinis_G05690 R_glutinis_G04646 R_glutinis_G00553 R_glutinis_G05814 R_glutinis_G03223 R_glutinis_G04524 R_glutinis_G03006	*crtO*	8
ketolase	R_glutinis_G03471 R_glutinis_G01624	*crtA*	2

## Conclusions

Here, the high-quality genome of *R. glutinis* ZHK was reported. It constructed a genetic basis for further research on its lipids, carotenoids, and industrial enzymes metabolism. Moreover, a total of 982 intracellular lipid species were identified in ZHK by lipidomic profiling, mainly including TAGs, DGTS and PE. In conclusion, our work provides new insights into these candidate genes and metabolites with potential biotechnological applications in *R. glutinis* ZHK. Furthermore, our results also lay the foundation for *R. glutinis* ZHK as a microbial cell factory to produce engineered compounds and facilitate the comparative genomics studies to elucidate evolutionary dynamics of the *Rhodotorula* species.

## Methods

### Yeast strain and growth conditions

In this study, the *Rhodotorula glutinis* strain ZHK was isolated from water of the Pearl River (23°06’N, 113°17’E), in Guangzhou City, Guangdong Province of China. Complete species identification was performed through morphology and molecular characterization, the GenBank Accession number of *R. glutinis* ZHK 26S rDNA is MT012072. The strain *R. glutinis* ZHK is cultured in 250 mL Erlenmeyer baffle flasks containing 50 mL of the YEPD medium (yeast extract: 5 g/L, peptone: 10 g/L, dextrose: 10 g/L, pH 6.5). After inoculation with the pre-cultured cell suspension of *R. glutinis* ZHK, cultures were transferred into flasks and incubated at 28°C on a rotary shaker at 180 rpm for 72 h. Fresh cells of *R. glutinis* ZHK were harvested by centrifugation (3000×g, 4°C, 10 min), immediately frozen in liquid nitrogen, and then stored at -80°C refrigerator for further extraction of genomic DNA, total RNA and metabolites.

### DNA extraction and genome sequencing

Genomic DNA was extracted using Universal Genomic DNA Kit (Cowin Bio., Beijing, China) according to the manufacturer’s instructions. The extracted DNA quality was detected using Qubit 2.0 Fluorometer (Life Technologies, USA) and Nanodrop (Thermo Fisher Scientific, USA) accordingly. The obtained genomic DNA with high-quality (≥ 100 ng/μL) was used for whole-genome sequencing through both the single molecule real time (SMRT) sequencing platform (Pacific Biosciences, USA) and the paired-end sequencing platform (Illumina, USA). Qualified genomic DNA was fragmented with G-tubes (Covaris, USA) and randomly end-repaired to prepare SMRTbell DNA template libraries (with an average fragment size of 30 kb) according to the manufacturer’s specification. Library quality was detected by Qubit 2.0 Fluorometer and average fragment size was estimated on a Bioanalyzer 2100 (Agilent Technologies, USA). SMRT sequencing was performed on the PacBio Sequel sequencer according to standard protocols (MagBead Standard Seq v2 loading, 1×180 min movie). Subsequently, the qualified genomic DNA was also used to construct a paired-end library with insert sizes of 300 bp using the Paired—End Genomic DNA Sample Prep Kit (Illumina, USA). These paired-end libraries were sequenced using the Hiseq 2500 sequencer with PE150 strategies according to the manufacturer’s protocols.

### Genome assembly using PacBio and Illumina data

Firstly, the PacBio long-reads was corrected for random errors in the long seed reads by aligning shorter reads from the same library using the program MECAT (https://github.com/xiaochuanle/MECAT) (version 1.0) as described previously [53, 54]. The corrected long reads were used for *de novo* genome assembly to generate contigs using the mecat2canu module of MECAT with an overlap-layout-consensus (OLC) strategy (parameters: min overlap length = 500; min read length = 1000) [53, 55]. After that, the Illumina paired-end short reads were used for polishing the resulting contigs through the program Pilon (version 1.22) with default parameters [56]. Finally, these polished contigs were further assembled to generate scaffolds using genomic synteny analysis (Additional file 10) with the closely related species *Rhodotorula graminis* [57]. Genome completeness of assembly was evaluated using the program BUSCO (version 3.0.1) with parameters: reference gene set of basidiomycota_odb9 [58].

### Phylogenetic and syntenic analysis

Phylogenetic analysis was performed by comparing the *R. glutinis* ZHK 26S rDNA sequence to other related species of the order Sporidiobolales. All 26S rDNA sequences were obtained from the NCBI Nucleotide database (https://www.ncbi.nlm.nih.gov/nuccore). MEGA 7.0 was used for processing these 26S rDNA sequences using Muscle alignment with UPGMB clustering method [59]. Phylogeny was tested using Bootstrap method with default parameters. Bootstrap values expressed as percentages of 1,000 replications were shown at the branching points. Phylogenetic tree was constructed using these aligned sequences by the Neighbor-Joining method in the software MEGA 7.0. Candidate species for syntenic analysis were selected based on the results of phylogenetic analysis. Syntenic analysis between the genome sequences of *Rhodotorula* species were performed by the program MCScanX with default parameters [60].

### RNA extraction and transcriptome sequencing

The extraction of total RNA was achieved with the TRIzol Kit (Invitrogen, USA). RNA quality was evaluated using the Bioanalyzer 2100 (Agilent Technologies, USA) and agarose gel electrophoresis (RNase-free) [61]. The high-quality RNA samples were used to constructed cDNA libraries for transcriptome sequencing. The workflow of cDNA library construction was performed as descripted previously [61, 62]. After that, the cDNA library was sequenced using Hiseq 2500 sequencer (Illumina, USA) to generate 150 bp paired-end reads. Raw reads were processed and further filtered through the FASTP program (version 0.18.0) to remove reads with following characters [63]: 1) containing adapters; 2) containing more than 10% of unknown nucleotides (N); 3) containing more than 50% of low quality (Q value ≤ 20) bases. After that, a reference genome index was built, and these paired end clean reads were mapped to the assembled *R. glutinis* ZHK genome using the program HISAT (version 2.2.4) with parameters: –rna-strandness RF and others set as default [64]. Mapped reads were assembled by using the software StringTie (version 1.3.1) in a reference-based approach [65]. For each transcription region, we used the software StringTie (version 1.3.1) (with default parameters) to quantify expression abundance and variations using a fragment per kilobase of transcript per million mapped reads (FPKM) method [66].

### Gene prediction and functional annotation

The open reading frame (ORF) was predicted using the program GeneMark-ES with default parameters (Additional file 11) [67]. Integration of mapped RNA-seq reads with genomic data to predict gene models was performed using the program GeneMark-ET with default parameters [68]. After using the program Stringtie to reconstruct these transcripts, the genes were expressed in the RNA-seq results but not included in the genome to be defined as the novel genes. Repetitive elements were identified by RepeatMasker (version v4.0.7) with default parameters [69]. Noncoding RNAs, such as rRNAs prediction was carried out using the program rRNAmmer (version 1.2) with default parameters and tRNAs were identified by the program tRNAscan-SE (version 2.0.4) with default parameters [70]. Functional annotation of the predicted genes was performed by aligning with diverse public databases [71], such as National Center for Biotechnology Information (NCBI) non-redundant protein (Nr) database, UniProt/Swiss-Prot, Kyoto Encyclopedia of Genes and Genomes (KEGG), Gene Ontology (GO), Cluster of Orthologous Groups of proteins (COG), and Protein families (Pfam) (Additional file 12).

### Identification of orthologous genes

Annotation information of coding sequences and proteins of *R. graminis*, *R. diobovata*, *R. toruloides*, and *R. taiwanensis* were downloaded from the NCBI Genome database (https://www.ncbi.nlm.nih.gov/genome/). Genome sequence alignments were performed in an all-against-all comparison using the MUMmer 3 package (http://mummer.sourceforge.net/, version 3.2.2) with default parameters [72]. Comparative genomics analysis of this study was performed at protein level. Software OrthoMCL (version 2.0) (https://orthomcl.org/orthomcl/) was used to generate core-orthologs for the *R. glutinis* ZHK, *R. graminis*, *R. diobovata*, *R. toruloides*, and *R. taiwanensis* proteomes datasets with default parameters [73]. Subsequently, all the putative proteins of the five yeast species and core-orthologs were aligned (all against all) using BLASTP (http://www.ncbi.nih.gov/BLAST/) and a score for each pair of proteins with significant matches was assigned with a cut-off value of 1×10^−7^ . GO and KEGG enrichment analyses of orthologous and species-specific genes were performed using DAVID functional annotation tool (https://david.ncifcrf.gov/tools.jsp, version 6.8) with default parameters [74].

### Substitution rate estimation

The substitution rates Ka/Ks (the number of nonsynonymous substitutions per nonsynonymous site to the number of synonymous substitutions per synonymous site) were averaged for all of the pairwise comparisons of each single-copy orthologous gene, using the free ratio Model of the software KaKs_Calculator Toolbox (version 2.0) with default parameters [75, 76]. These genes showed a higher Ka/Ks value (Ka/Ks > 1 or 0.5 and *p*-value < 0.05) were suggested to be the positively selected genes [77].

### Lipidomics analysis

To perform the lipid profiling, the methyl tert-butyl ether (MTBE) method was used for lipid metabolites extraction [78]. Briefly, there are about 1×10^7^ cells from three growth periods (24 h, 48h, and 72h) of each sample, which was added to 450 μL of extraction liquid (V_MTBE_: V_methanol_ = 5:1) and further vortexed for 30 s. The mixture was then centrifuged at 3000×g, 4°C, for 15 min. The supernatant (organic layer) was transferred to a clean vial and evaporated to dryness in a vacuum concentrator. Dried extract was reconstituted in 100 μL dichloromethane/methanol liquid (1:1, v/v). 60 μL liquid extract of each sample was collected for the following analysis. Lipid metabolites profiling was performed by an Ultra-high-performance liquid chromatography system (UHPLC 1290 series, Agilent Technologies, USA) with a C_18_ column (1.7 μm, 100 mm×2.1 mm, 100 A) (Phenomen Kinetex, USA) coupled with a quadruple time-of-flight mass spectrometer (Triple TOF 6600, AB SCIEX, USA). The acquisition software Analyst TF (version 1.7.1, AB SCIEX, USA) continuously evaluated the full scan survey MS data with default parameters. Detailed settings of the UHPLC-MS system were used as described previously [79]. Raw data files acquired from UHPLC-MS/MS were transformed into the mzXML format using the program ProteoWizard (version 3.0.4472) with default parameters [80], and further analyzed by R package XCMS (version 3.2) with default parameters [81, 82]. Peak annotation of the XCMS preprocessed data was performed using R package CAMERA with default parameters [82, 83]. Metabolites were identified through matching the acquired MS/MS spectral against the LIPID MAPS® (https://www.lipidmaps.org/) and an in-house standard MS/MS database (Biotree Biotech Co., Ltd., Shanghai, China) with the parameters (|m/z errors| <25 ppm, cutoff of match score=0.6, and minfrac=0.5) as described previously [79]. To ensure the accuracy of lipidomics data, the quality control (QC) samples were used for data evaluation [84]. All lipidomics analysis was performed with three independent biological replicates.

### Carotenoids extraction and quantification

Carotenoids were extracted at 65°C for 30 min from freeze-dried *R. glutinis* ZHK cells using DMSO (dimethylsulfoxide)-acetone (1/3, v/v) [49]. After centrifugation of 6000×g, the pigmented supernatant (organic layer) was pipetted off and the extraction was repeated until it became entirely colorless. High performance liquid chromatography (HPLC) analysis of carotenoids was performed on an Agilent 1100 series system (Agilent Technologies, USA). A reverse-phase C_18_ column (5 μm, 150×4.6 mm) (Thermo Fisher Scientific, USA) was used for the separation of carotenoid extracts. HPLC analysis in an isocratic elution system: acetonitrile-methanol-isopropanol (85:10:5, v/v/v) as the mobile phase at a column temperature of 32°C; an injection volume of 20 μL; a flow rate of 1.0 mL/min; the UV-visible spectra were obtained at 450 nm (for β-carotene), 484 nm (for torulene), and 507 nm (for torularhodin), respectively. Carotenoid quantification was performed with three independent biological replicates. Standard substance of β-carotene was bought from Sigma-Aldrich (St. Louis, MO, USA). Standards of torularhodin and torulene were purchased from Carote*Nature* GmbH (Münsingen, Switzerland).

## Supplementary Information

**Additional file 1.** Genome completeness analysis through aligning the orthologs of the ZHK to a reference gene set of basidiomycota_odb9.

**Additional file 2.** All genes expression profile resulted from RNA-seq analysis.

**Additional file 3.** SNP/InDel annotations resulted from RNA-seq data.

**Additional file 4.** Gene structure optimization resulted from RNA-seq data.

**Additional file 5.** All the genes involved in the biosynthesis of antibiotics in the ZHK genome.

**Additional file 6.** Comprehensive information of all identified lipid metabolites.

**Additional file 7.** Annotations of species-specific genes of the ZHK.

**Additional file 8. **One-to-one orthologous genes between *R. glutinis* ZHK and its close related species *R. toruloides*, *R. graminis*, *R. diobovata* and *R. taiwanensis.*

**Additional file 9. ***Ka*/*Ks* results of single-copy orthologous genes.

**Additional file 10. **Genomic synteny analysis between the ZHK and its closely related species *Rhodotorula graminis*.

**Additional file 11.** CDS/cDNA sequences resulted from genome analysis.

**Additional file 12.** Annotations of all gene resulted from assembled ZHK genome.

## Data Availability

All the raw sequence data are available via GenBank under the SRA accessions SRR11648405 (Illumina paired-end raw data of RNA-seq), SRR11637747 (Illumina paired-end raw data of genome sequencing) and SRR11611234 (PacBio long-read raw data of genome sequencing). The *R. glutinis* strain ZHK Whole Genome Shotgun project has been deposited at DDBJ/ENA/GenBank under the accession JAAGPT000000000. The version described in this paper is version JAAGPT010000000.

## Abbreviations

BP: Biological Process
CC: Cellular Component
GO: Gene Ontology
HPLC: High Performance Liquid Chromatography
KEGG: Kyoto Encyclopedia of Genes and Genomes
COG: Cluster of Orthologous Groups of proteins
MF: molecular function
MS: Mass Spectrum
Nr: NCBI's non-redundant protein database
Pfam: Protein Families
Rfam: RNA families
